# Comparative Methods for Molecular Determination of Host-Specificity Factors in Plant-Pathogenic Fungi

**DOI:** 10.3390/ijms19030863

**Published:** 2018-03-15

**Authors:** Nilam Borah, Emad Albarouki, Jan Schirawski

**Affiliations:** Institute of Applied Microbiology, RWTH Aachen University, Microbial Genetics, Worringerweg 1, 52074 Aachen, Germany; nilam.borah@rwth-aachen.de (N.B.); emad.albarouki@rwth-aachen.de (E.A.)

**Keywords:** host specificity, plant pathogen, fungi, effector, sequencing, genotyping

## Abstract

Many plant-pathogenic fungi are highly host-specific. In most cases, host-specific interactions evolved at the time of speciation of the respective host plants. However, host jumps have occurred quite frequently, and still today the greatest threat for the emergence of new fungal diseases is the acquisition of infection capability of a new host by an existing plant pathogen. Understanding the mechanisms underlying host-switching events requires knowledge of the factors determining host-specificity. In this review, we highlight molecular methods that use a comparative approach for the identification of host-specificity factors. These cover a wide range of experimental set-ups, such as characterization of the pathosystem, genotyping of host-specific strains, comparative genomics, transcriptomics and proteomics, as well as gene prediction and functional gene validation. The methods are described and evaluated in view of their success in the identification of host-specificity factors and the understanding of their functional mechanisms. In addition, potential methods for the future identification of host-specificity factors are discussed.

## 1. Introduction

The concept of host-specificity of plant-pathogenic fungi has always intrigued plant pathologists. Why can some fungal plant pathogens cause disease only in one specific host and not in others; or how can a fungal pathogen of a particular plant adapt and switch to a new host and thereby become a new pathogen for the new host, are questions that have shaped and will shape research in plant-fungus interaction. The term host-specificity refers to the capability of some fungal species or some members of one fungal species to cause disease only on particular plant species or only on some members of a specific plant species. Molecular models have been developed to explain the basis of host-specificity, like the gene-for-gene hypothesis developed by Henry H. Flor following his careful observations of the interactions of flax with flax rust [1]. According to this hypothesis, incompatible interactions result from the presence of resistance (R) proteins in the particular plants that recognize specific avirulence (AVR) proteins of the fungus, which results in successful plant defense against the pathogen. All interactions of plants carrying a particular R gene with pathogens carrying the corresponding *AVR* gene would be incompatible. This model has been refined by the guard and later the decoy models that predict that the AVR-R interaction is indirect, with the R protein guarding a target of the AVR protein, or guarding a decoy, a functionally inactive version of the target [1,2,3]. These models have led to a tremendous increase in the understanding of plant-pathogen interactions. The second great advancement in this understanding is the discovery of effectors—small, secreted, variable, genome-encoded proteins of the pathogens—that are essential to modulate the plant response [3,4,5]. The realization that effectors can have virulence and/or avirulence (AVR) functions elegantly links the two concepts and explains the multitude of observed species-specific effector genes on the pathogen side as well as the multitude of receptor genes with the role of R proteins on the plant side, by an evolutionary arms race [5,6].

Is there a difference between host specificity factors and virulence factors? The answer is: yes and no. It is yes, because some virulence factors are essential for basic and conserved virulence functions that are necessary for the processes of plant infection. These basic virulence factors, although essential for virulence of host-specific pathogens on their preferred host plants, do not contribute to incompatibility with the related plants and are, therefore, not involved in determining host specificity. However, the answer is also no, because all host-specificity factors should modulate virulence of the pathogen by either contributing to avirulence on the non-preferred or to virulence on the preferred host, or both. This means that all host-specificity factors should be virulence factors, but not all virulence factors are necessarily host-specificity factors.

Are all host-specificity factors effectors? Here the answer is no. While some host-specificity factors are indeed effectors, like for example the PWT3 and PWT4 effectors of *Magnaporthe oryzae* [7], the classical and best-known host-specificity toxins of *Alternaria alternata* are secondary metabolites generated by polyketide synthases (PKSs) [8,9,10]. The *PKS* genes reside on a conditionally dispensable chromosome, and transfer of the chromosome containing the PKS necessary for virulence of *A. alternata* tomato pathotype to a strawberry pathotype leads to strains able to produce both toxins needed for infection of tomato and strawberry [11].

While we have a fairly good understanding of host-adaptation in some systems, in others the mechanism is still unknown. Even if the mechanism can be inferred, identification of the participating factors is still quite challenging. For example, after knowledge that the *Ustilago hordei*-barley interaction is governed by AVR-R gene interactions, it took 32 years to clone an 80-kb region containing *AVR1*, and an additional 10 years to identify the gene encoding AVR1 [12,13,14]. Therefore, powerful methods are needed for the identification of host-specificity factors to unravel the molecular basis of host adaptation of phytopathogenic fungi to their plant hosts.

**Figure 1:**
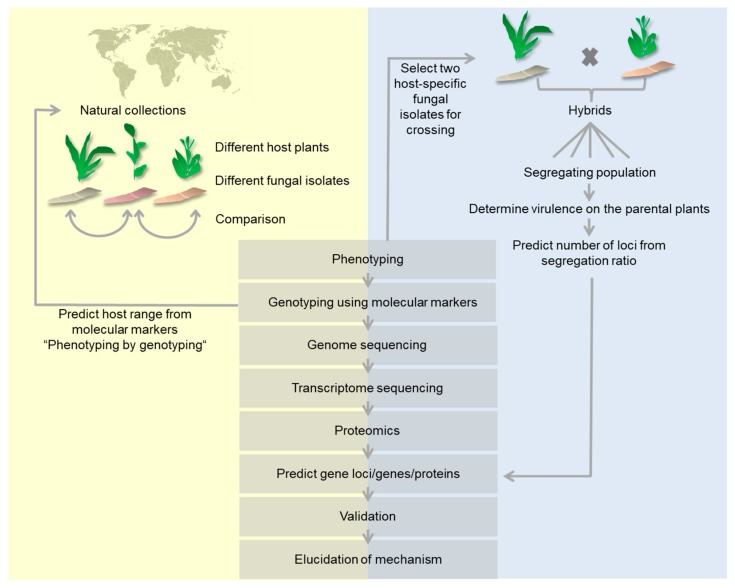
Graphical representation of comparative methods used to determine molecular host-specificity determinants. Methods starting with a collection of natural fungal isolates (yellow background) are contrasted to methods starting with segregating populations of defined strains (gray background). Both starting materials use the same set of analyzing methods.

In the following, we review molecular methods that have been employed for the determination of host-specificity factors. We focus in this review on comparative analyses of two or more different pathovars, *formae speciales*, isolates, races or species of plant-pathogenic fungi. Where appropriate, we also included examples from oomycetes. We excluded from our review studies that investigate the contribution of individual factors to virulence on one host, even if these factors may be host-specificity factors. We also largely excluded literature on host-specificity of symbiotic mycorrhiza in spite of their importance, economic relevance and recent success in the identification of effectors necessary for plant infection [15]. We present the methods employed in chronological order, starting with methods for definition and characterization of the pathosystem, methods for genotyping, comparative -omics techniques like genomics, transcriptomics and proteomics, gene prediction and functional validation (Figure 1).

These methods are explained and evaluated in view of their success in the identification of host-specificity factors and the understanding of their functional mechanisms. For a review of known host-specificity mechanisms and factors, we would like to draw the reader’s attention to previous reviews [16,17].

## 2. Defining and Characterizing the Pathosystem

Any comparative molecular analysis is dependent on a well-characterized pathosystem. It is, therefore, not astounding that many studies collected and phenotypically characterized the available biodiversity of natural fungal isolates. Fungal isolates are collected from different parts of the world e.g., from fields of host plants that are infected by the fungus [18,19], or procured from various laboratories and culture collections [20]. Depending on the necessity and type of infection, fungal specimens can be isolated from any part of the plant, including infected leaves, stems or inflorescences [21]. After collection, the natural isolates are phenotypically characterized either by determining infection capability on different host plants coupled with microscopic analysis and analysis of the disease phenotype [22,23,24,25,26,27,28,29,30,31,32,33,34], or by the characterization of physiological traits, like their ability to produce or degrade various chemicals [29,31,35,36]. This way, various pathosystems were defined, like the *formae speciales* of *Fusarium oxysporum*, of *Puccinia*, and of *Alternaria*.

A well-defined pathosystem is the basis of any follow-up comparative analysis. From this pool of data, two different approaches are followed. In one approach, two isolates with different host-specificity are crossed or hybridized, generating a segregating population with differential capabilities to infect one or the other host plant. This segregating population is then analyzed to define genomic loci associated with differential infection capabilities. In the other approach, the complete pool of natural isolates is used for phenotypic and molecular analyses to link genomic markers to phenotypic traits (Figure 1). The approach using segregating populations generated by hybridization of host-specific isolates will be treated in a separate section below. In most examples, however, the approach using natural populations was followed.

## 3. Using Molecular Markers for Genotyping

To link genomic loci or markers to the host infection-specificity phenotype, many different methods have been developed that allow a molecular comparative characterization of the different isolates. These methods include analysis of restriction fragment-length polymorphism (RFLP), amplified fragment-length polymorphism (AFLP), random amplified polymorphic DNA (RAPD), micro- and minisatellites, mitochondrial haplotype, internal transcribed spacer (ITS) regions, as well as of complete proteomes and genome sequences. The advantages and disadvantages of these methods are summarized in Table 1. Examples of successful use in the genomic characterization of different fungal isolates are presented below.

A restriction fragment-length polymorphism (RFLP) is existent if a base substitution in the genomic DNA occurs within a restriction enzyme-recognition sequence leading to differently sized restriction fragments. Because a digest of genomic DNA with a restriction enzyme leads to too many restriction fragments to visualize individual length polymorphisms, the use of labeled probes hybridizing to specific regions of the genomic DNA is necessary. The probes may cover mating type genes [37], host-species specific repetitive DNA sequences of the pathogen [38], or fungal isolate-specific genomic regions [39]. The use of telomere-linked RFLP revealed a specific pattern for almost every strain of *Botrytis cinerea* isolated from different host plants from various regions, which showed the presence of extended polymorphism near telomeres in *B. cinerea* [40] and was, therefore, too specific to link a telomere-RFLP to the host infection phenotype. Identical sequence at the internal transcribed spacer (ITS) regions of the genes encoding ribosomal RNAs is used to verify close genetic relationship between isolates [41,42] but can also be used to differentiate host-specific fungal isolates. An ITS-based RFLP–polymerase chain reaction (PCR) method was successful in distinguishing different barley- and rye-infecting isolates of *Rhynchosporium* [43]. RFLP analysis using the 18–28S ribosomal transcriptional unit from *Aspergillus nidulans* as the probe revealed similar RFLP patterns among all pink and gray *Colletotrichum* isolates regardless of host origin. However, RFLP patterns of pink isolates were distinct from those of gray isolates [24]. This method was thus shown to be useful to differentiate two isolates based on initial morphological characterization, later supported by molecular marker polymorphism. Mitochondrial DNA (mtDNA) RFLP by *Hae*III-digestion of total genomic DNA was used to efficiently differentiate two *formae speciales* of the *Fusarium oxysporum* species complex, f. sp. *lycopersici* (Fol) and f. sp. *radicis-lycopersici* (Forl). These two *formae speciales*—although morphologically identical and capable of infecting the same tomato cultivar—cause distinct disease phenotypes [44]. PCR amplification of rDNA ITS regions followed by enzymatic digestion to perform RFLP has also been used to study host preferences of oomycete species within ecologically contrasting sites [45]. RFLP fingerprinting of oomycete isolates was used effectively in *Phytophthora infestans* to differentiate different clonal lineages [46,47]. From these examples it becomes clear that while RFLP can be used to differentiate specific isolates, the use of probes drastically limits the investigated genomic area so that the identification of an RFLP pattern that correlates with host-specificity depends on serendipity.

A genome-wide and less biased comparative approach is the use of amplified fragment length polymorphism (AFLP) [48]. In this technique, the genomic DNA is first digested with two different restriction enzymes, a frequent cutter and a rare cutter. Then specific adaptors are ligated that allow the PCR-amplification of restriction fragments. The number of amplified restriction fragments is reduced by extending the primers by 1 or 2 nt into the restriction fragment. To further reduce the number of amplified restriction fragments, a second PCR with labeled primers follows that extend by 3 to 4 nt into the restriction fragment. In this PCR, hybrid fragments with one rare-cutter adaptor and one frequent-cutter adaptor are preferentially amplified because two frequent-cutter adaptor primers form secondary structures that hamper product amplification. Primers with different extensions are tested to obtain about 50 to 120 amplified fragments, whose template-dependent lengths can then be compared by gel electrophoresis and label detection. AFLP has been successfully used to differentiate race composition, race variation and host-specific isolates, such as *P. infestans* isolates collected from cultivated potatoes and the native wild *Solanum* spp. *Solanum demissum* and *Solanum xendinense* in the Toluca Valley of central Mexico, and *Magnaporthe grisea* isolates associated primarily from perennial ryegrass and kikuyugrass in golf courses in California [21,49,50]. Host-specific groups could also be defined according to the AFLP pattern of various endophytic fungi following initial morphological identification [51]. *Blumeria graminis* has been classified into eight *formae speciales* with different host specificity. rDNA ITS region and β-tubulin gene-based phylogenetic analysis showed grouping of isolates according to their principal host genus, which was further supported by AFLP analysis [52]. AFLP data can also be used for the generation of phylogenetic analysis, revealing the origin of host-specific taxa [20].

A special form of AFLP is the investigation of the mitochondrial DNA (mtDNA) haplotype. In this technique, certain regions in mitochondrial genomes are first PCR-amplified and then subjected to restriction-enzyme digestion. mtDNA haplotype analysis has been successfully used to characterize different isolates of *P. infestans* [47,50]. The result can reveal whether the population structure is of monomorphic or polymorphic nature. The mtDNA haplotype has also been investigated in various geographical isolates of *Colletotrichum orbiculare* having a common host and in various other species of *Colletotrichum* [53]. The data helped to link host specificity with the haplotype pattern, thereby suggesting a way to recognize different host-specific isolates.

Random amplified polymorphic DNA (RAPD) is a PCR-based method that does not rely on restriction enzymes. A 10 nt primer of arbitrary sequence is used to amplify random segments of genomic DNA. This method has been widely used to identify and isolate molecular markers specific to a particular fungus, thereby helping in diagnosis of fungal infection in symptomless plants [54,55]. RAPD was used to characterize the genetic differentiation and correlation with host specificity among *Alternaria* spp. that cause brown spots on different *Citrus* spp. [26,56]. In *F. oxysporum*, a robust RAPD protocol was developed to identify economically important strains infecting specific hosts [57]. The RAPD technique was also used to discriminate between isolates of different host-specialized *Rynchosporium* species [58]. In *U. hordei*, RAPD was used in combination with microsatellites (see below) to assess genetic variation among different isolates in Tibetan areas of China [59]. In addition, RAPD has also been used as a tool to differentiate *formae speciales* of *Microbotryum violaceum* and to characterize the genetic diversity of the Italian population of *Ceratocystis fimbriata* f. sp. *platani* [60,61]. Although RAPD is a more unbiased technique covering a larger part of the fungal genome, the method has issues with reproducibility. Therefore, it is usually only used in combination with other markers, like micro- or mini-satellites.

Satellite DNA was first observed as a less-dense band of DNA clearly separated from the bulk of chromosomal DNA during density-gradient centrifugation. Microsatellites or simple sequence repeats (SSR), and their longer cousins, the minisatellites, consist of AT-rich repetitive DNA that seem to have a higher mutation rate resulting in a larger genetic diversity than other genomic regions. This genetic diversity can be used to discriminate different fungal isolates by PCR-amplifying the repetitive DNA [62]. Microsatellites have been found to be more informative for genotyping isolates from different hosts of the necrotrophic fungus *Botrytis cinerea* than RFLP patterns of the ADP-ATP translocase and nitrate reductase genes or MSB2 minisatellite sequence data [63]. In a study of the anther smut fungus *M. violaceum*, genetic diversity in sympatric, parapatric and allopatric populations of two host species was found using four polymorphic microsatellite regions [64]. In *Pyrenophora semeniperda*, that was shown to lack host specialization, weak yet significant population genetic structures as a function of host species could be observed by the use of seven polymorphic microsatellite loci [34]. Thus, the investigation of the diversity in microsatellite loci is a very sensitive tool to discriminate between different fungal isolates and to visualize even weak associations. However, microsatellite diversity has not yet been shown to be causally related to host specificity.

Other repetitive elements like retrotransposons or transposable elements have also been used for the characterization of host-specific fungal isolates. When using the reverse transcriptase gene of the LTR-retrotransposon *CfT-l* from *Cladosporium fulvum* as a probe, different *formae speciales* of *F. oxysporum* could clearly be differentiated [65]. PCR-detection of two transposable elements revealed different population structures of *B. cinerea* on a variety of different host plants [66].

**Figure 2:**
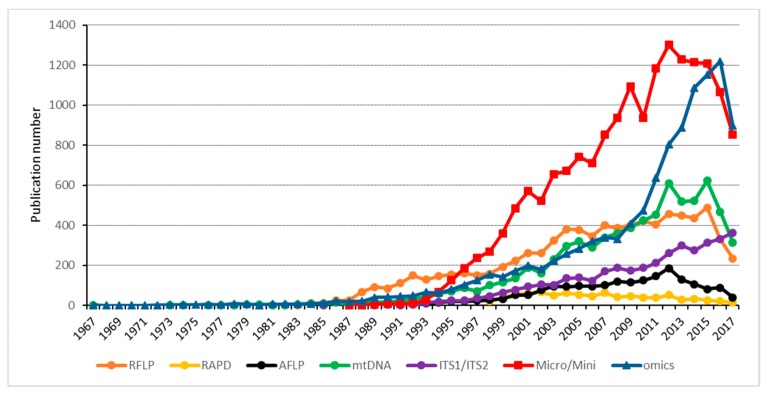
Hits of the search terms in the PubMed data base sorted by year of publication between 1967 and 2017. The -omics techniques, despite their relatively recent appearance and relatively high cost in comparison to other techniques, are increasingly popular. Analysis of micro- and minisatellites is still popular. The search terms were as follows: “Restriction fragment length polymorphism, RFLP analysis AND population genetic” for RFLP, “RAPD analysis AND population genetic“ for RAPD, “Amplified fragment length polymorphism; AFLP analysis AND population genetic” for AFLP, “mitochondrial DNA; mtDNA; mitochondrial DNA Analysis AND population genetic” for mtDNA, “ITS1/ITS2; ITS1 OR ITS2 analysis“ for ITS1/ITS2, “Micro/Minisatellite repeats OR micro/minisatellite analysis AND population genetic“ for Micro/Mini, and “High throughput nucleotide sequencing, OR next generation sequencing analysis AND population genetic” for omics.

It seems that a large number of different techniques have been developed that allow for molecular comparison of different organisms. Of the described techniques, AFLP and microsatellites seem to be the most sensitive methods to molecularly discriminate fungal isolates for which no or only very little sequence information is available. However, most studies using these methods did not come up with a clear genomic link to host-specificity of the investigated fungal strains. This means that other, even more precise techniques are needed to decipher the molecular basis of host-specificity. Here, the field has profited tremendously from the development of the next-generation sequencing (NGS) techniques that now allow cost-effective genome sequencing and data assembly of large fungal genomes. Since they have become available, the use of -omics (genomics, transcriptomics, and proteomics) approaches highly outnumber the use of the classical comparative approaches described above (Figure 2).

## 4. Comparing Whole Genome Sequences

One of the first eukaryotic genomes sequenced using next-generation sequencing techniques was that of *Sporisorium reilianum* f. sp. *zeae*, a close relative of *Ustilago maydis*. Both cause smut disease of maize but induce quite distinct symptoms. Genome comparison revealed that both genomes were highly syntenic but contained so called “divergence clusters” containing genes with below-average sequence conservation between the two organisms [67,68]. Within these divergence clusters, a high percentage of genes encoded proteins containing predicted secretion signal peptides and were thought to be involved in interaction with the plant, thereby explaining their increased evolution rate. Deletion analysis of complete cluster regions in *U. maydis* confirmed the role in virulence for four of six randomly selected clusters [67,68]. Genome sequencing of the related barley smut pathogen *U. hordei* allowed comparison with an organism showing a similar infection strategy as *S. reilianum* and being virulent on a different host plant. The three-way genome comparison revealed among others that most of the weakly conserved genes present in the *S. reilianum*/*U. maydis* divergence clusters have weakly conserved homologs in *U. hordei* [67,68], which supports the proposition that these proteins could play a function in adaptation to different hosts or lifestyles. Sequencing of the *Sporisorium scitamineum* genome allowed a four-genome comparison of effector genes [69]. This comparison revealed that evolution of effector-encoding clusters is driven by tandem gene duplication and the activity of transposable elements, and supports the conclusions drawn from analysis of the *U. hordei* genome [67,68,69]. Thus, genome comparison of smut fungi so far has resulted in lists of genes potentially encoding host-specificity factors.

Interesting gene candidates with a suspected role in host-specificity were also obtained when comparing the genomes of the closely related species *Colletotrichum graminicola* and *Colletotrichum sublineola* [70]. The main differences of the otherwise very similar genomes were found in genes for biosynthesis of specialized secondary metabolites and of small secreted protein effectors. However, whether these genes indeed contribute to host selection has yet to be tested. Key enzymes of fungal secondary metabolism and effector proteins were identified as potential host-specificity factors by genome comparison of *Rhynchosporium* species [71], and of members of the *Fusarium fujikuroi* species complex, where species-specific and isolate-specific differentiation in secondary metabolite-producing genes both in composition and expression were detected [72]. The comparison of whole genome sequences of four different strains of the *C. acutatum* species complex showed that changes in gene content were related to changes in host range with lineage-specific gene losses and gene-family expansions [73]. Gene loss was also suggested as a cause of fungal adaptation to a new dicot host after a host jump from a monocot plant by the smut fungus *Melanopsichium pennsylvanicum*. When compared to the genomes of three other smut fungi, *M. pennsylvanicum* was found to lack putative effector genes [74]. In addition to putative effectors, comparative whole genome and transcriptome analyses of *Lasiodiplodia theobromae* and five other *Botryosphaeriaceae* pathogens causing opportunistic infections in woody plants identified two in-planta expressed lignocellulose genes, whose overexpression increased virulence of the pathogens [75].

In a genome comparison study of the three species *Fusarium graminearum*, *Fusarium verticillioides* and *F. oxysporum* f. sp. *lycopersici* (Fol) lineage-specific (LS) genomic regions and LS chromosomes were discovered [76]. The authors of the study could prove experimentally that the presence of Fol LS chromosome 14 provides specificity for *Fusarium* adaptation towards the tomato [76], limiting the search for host-specificity factors to a single chromosome. This information was used in a recent study where the genomes of three legume-infecting *formae speciales* of *F. oxysporum* were compared to the genomes of the tomato-infecting Fol and the pea-infecting *Fusarium solani*. Combining comparative genome analysis with predicted LS gene content and *in-planta* transcription analysis revealed four candidate effectors conserved among legume-infecting *formae speciales* [77]. Clustering of presence/absence patterns of candidate effector genes following whole genome sequencing of five different *formae speciales* of *F. oxysporum* showed clustering of members of the same *forma specialis* [78] suggesting that effectors contribute to host specificity. This confirms and extends earlier reports of an association of the three *secreted in xylem* (SIX) effectors SIX1, SIX2 and SIX3 with tomato-infecting isolates [79], and of the suitability of the presence/absence determination of *SIX1* to *SIX5* as a robust method to differentiate different *formae speciales* [80].

For the identification of effectors, sequencing of RNA or cloning of expressed sequence tags (ESTs) can be very helpful. Sequencing of about 2000 cloned ESTs from *C. lentis*-infected lentil leaf tissues enabled annotation of 15 candidate effectors. Infection stage-specific gene expression was observed for the candidate effectors. One candidate effector, CICE6, was found to carry a single nucleotide polymorphism (SNP) that could be used efficiently to differentiate between two pathogenic races of *C. lentis* [81]. Comparison between transcriptional profiles of two races of *F. oxysporum* f. sp. *cubense* revealed a remarkably different gene-expression profile in response to a host cell wall [82] suggesting that differences in gene expression could contribute to host-specificity.

Without being exhaustive, this enumeration already shows that the field has profited tremendously from the development of next-generation sequencing techniques. While the classical methods described above using molecular markers have been mostly used for the characterization of host-specific isolates, no precise gene candidates have resulted. In contrast, genome comparison that is sometimes coupled to a comparison of the transcriptional profile, has often resulted in the identification of genomic regions, and sometimes even of genes that are associated with adaptation to a specific host. These genomic regions and these gene lists, therefore, contain strong candidates for the determination of host-specificity. It seems that the methods depending solely on molecular markers are not precise enough to result in the prediction of genes associated with a certain host-infection phenotype. Novel methods, like diversity array technology sequencing (DArTseq) have been developed that combine mapping technology with next-generation sequencing. In DArTseq, different restriction enzymes are used to cut the genomic DNA prior to sequencing, which separates the low-copy number DNA from repetitive sequences that are cut to small pieces. The combination of precise mapping of the avirulence phenotype on a certain wheat cultivar with gene-expression data in infected plant tissue resulted in the successful identification of AvrStb6 as responsible for avirulence of *Z. tritici* IPO323 on wheat cultivar Shafir [83]. Therefore, when aiming at identifying the responsible genes for host-specific infection, it is much more promising to resort to genome sequencing, to do genome comparison and to couple this data to the analysis of gene-expression profiles.

## 5. Comparing Complete Proteomes

An alternative approach to genome comparison and transcriptome analysis for the identification of proteins involved in host-specificity determination is the direct comparative analysis of the fungal proteomes. Several studies have compared the proteomes of different isolates or species with the idea of identifying proteins crucial for host-specificity. Tandem mass spectrometry was used as a tool to compare the proteomes of hyphae and germinating cysts of two closely related oomycetes, *Phytophthora pisi* and *Phytophthora sojae*, that cause disease on pea and soybean, respectively [84]. A global proteomic comparison of mycelium and germinating cysts was done in two other oomycete plant pathogens, *Phytophthora ramorum* and *P. sojae* [85]. A proteome comparison was also done with uredospores from two different populations of the rust fungus *Puccinia psidii* isolated from eucalyptus leaves and guava fruits [86]. Mycelial proteins from isolates of the brown rot fungus *Monilinia laxa* were obtained from apples and apricots, and were separated by 2-D gel electrophoresis, followed by LC-MS/MS of identified differentially expressed proteins [87]. In all of these studies, clear differences in the fungal proteomes were observed, and detected species- or population-specific proteins were suspected to have a role in host adaptation. However, the lists were long, and a causal connection of identified proteins to host specificity has not yet been shown.

One problem of comparing proteomes of infected plant material is that a high number of plant proteins may be differently expressed or differently modified because the two investigated fungal strains behave differently on the plant, rather than the different plant protein profiles being the reason for the different behavior of the fungal strains. Therefore, identifying fungal determinants of host-specific proliferation will be a difficult task to solve by proteome comparison alone.

Global comparative approaches have brought the scientific community much closer to the goal of identifying host-specificity factors by providing lists of genes or proteins that have a high probability of being involved in host adaptation. The follow-up of these approaches would now consist of testing individual high-probability candidates for their specific contribution to host selection. This can be challenging, especially if genetic tools for the investigated system are not yet available, or if lists contain several dozens of putative candidates. Success of the follow-up experiments will depend a great deal on the predictive quality of these lists, which will increase with closer relatedness of the compared pathogen genomes.

## 6. Investigating Segregating Populations

One way to overcome the problem of having to test candidates from long lists of putative host-specificity factors that have a certain possibility of being involved in host adaptation, is to investigate a well-defined population that segregates for the host-infection phenotype. The easiest method to obtain such a well-defined population is to generate it by targeted hybridization experiments or genetic crosses of closely related fungal isolates that differ in their infection phenotype.

Hybrids have been investigated before the knowledge of genome sequences. Mating compatible isolates of *Colletotrichum gloeosporioides* with different host specificities were crossed, and ascospore offspring were analyzed for virulence and RFLP patterns. However, no correlation between pathogenicity to the parental hosts and presence/absence of any RFLP marker could be found [88]. In a comparative study of various host-specific *Ascochyta* species, interspecific crosses could be obtained, and offspring analysis showed that AFLP markers segregated freely. While not being able to assign specific AFLP markers to host infection capacity, it was speculated that host-specificity may contribute to speciation [28]. F1 progeny of a cross of two *Leptosphaeria maculans* isolates that differed in their capacity to cause disease on *Brassica juncea*, were used to create a genetic map of different AFLP and RAPD markers as well as the mating type and a host-specificity locus that could be placed on the end of chromosome 9 [89]. Hybrids of *M. oryzae* isolates from rice and wheat were generated and the resultant F1 population was tested on wheat for pathogenicity [90]. The segregation ratio of avirulent to virulent offspring provides information of the number of loci involved. In this case, a ratio of 7:1 led to the prediction of three loci being involved in avirulence of the *M. oryzae* rice isolate on wheat. Allelism tests could associate two known loci, Pwt2 to papilla formation and Pwt1 for hypersensitive reaction. The third locus did not correlate with any known loci and was, therefore, named Pwt5 [90]. In a F1 population of a cross between wheat- and foxtail millet-pathogenic *M. grisea*, virulent isolates segregated in a 1:1 ratio on foxtail millet cultivars Beni-awa and Oke-awa but not on cultivar Kariwano-zairai, suggesting that specificity of *M. grisea* toward foxtail millet is governed by cultivar-dependent genetic mechanisms like gene-for-gene interactions [91].

**Figure 3:**
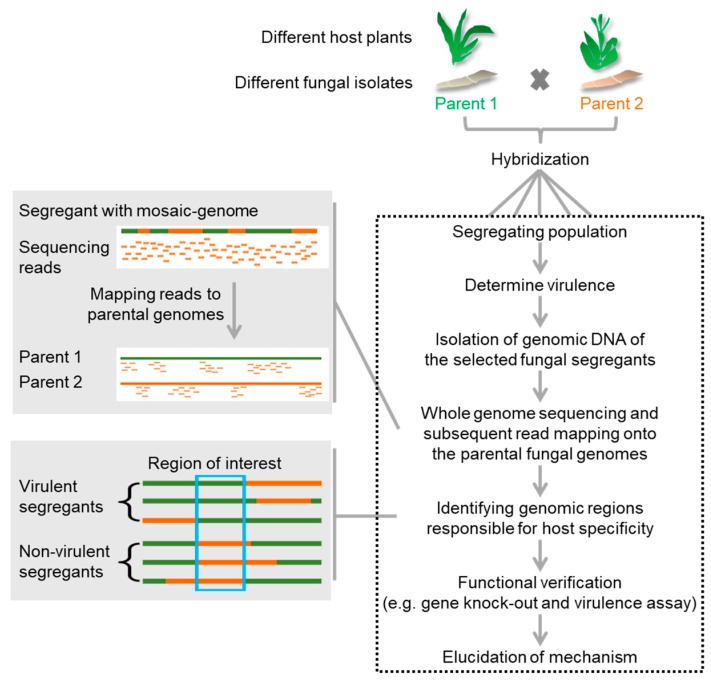
Strategy for identification of host-specificity factors using segregating populations of a hybridization event between two host-specific individuals. Virulence capacity of each offspring on one or both hosts is individually determined. Genome sequencing of fully virulent and avirulent offspring reveals parental origin of the mosaic genomes. Associating the parental origin of specific genomic loci to the virulence phenotype should lead to identification of genomic regions linked to host-specific virulence.

Since these studies were all conducted without the knowledge of the genome sequences, it is conceivable that the combination of genome sequencing and the investigation of defined segregating populations will either allow much faster genome map-based cloning or, through the sequencing of complete populations, allow high-resolution single nucleotide polymorphism (SNP) mapping of host-specificity traits. Creating genetic linkage maps in fungi is an underdeveloped but potentially important field that is expected to gain future attention in the light of whole genome sequencing [92]. In this light, we have started to analyze hybrids of two compatible *formae speciales* of the head smut fungus *S. reilianum*, *S. reilianum* f. sp. *zeae* (SRZ) and *S. reilianum* f. sp. *reilianum* (SRS) that either infect maize (SRZ) or sorghum (SRS). On each other’s hosts, both fungi can colonize but do not cause smut disease [93]. A population of meiotic progeny (SRSZ) of a mating event between SRS and SRZ was generated and for each individual strain the virulence potential on sorghum was tested and varied greatly between individuals (unpublished). To associate the virulence phenotype on sorghum to particular genomic-regions, about 190 strains were selected for genome sequencing. Mapping of sequencing reads to the parental genomes confirmed the presence of mosaic genomes in the offspring (unpublished). A detailed genome analysis will show whether specific parental genomic regions can be associated with the virulence phenotype on sorghum (Figure 3). As soon as such an associated region is known, knowledge of the genome sequence will allow direct prediction of candidate genes for validation of their role in determining host-specificity.

## 7. Validating Functional Contribution to Host-Specificity

Generating lists of the best potential candidates for involvement in host-specificity determination is already a great step forward. However, the goal is to show that a given candidate has indeed a role in host-specificity. Validation of a functional involvement of a specific candidate gene could be done by the generation of gene deletion or overexpression strains and monitoring of the mutant’s host preference. As mentioned above, this can be a challenging task. In a few cases, functional validation was done and showed a clear relationship of the identified genes with host-specificity.

In many cases, genes involved in host-specificity were identified as a part of classical AVR-R gene interactions. For example, using crosses of *M. grisea* strains infecting different grass species, single genes that determine specificity towards the host weeping lovegrass (*Eragrostis curvula*) have been identified [94] and were shown to be part of a gene family [95]. The inability to infect weeping lovegrass could be associated with one member of the gene family, *PWL2*, where frequently occurring loss-of-function mutations of *PWL2* led to spontaneous pathogenic mutants [96]. Thus, *PWL2* has all the characteristics of a classical avirulence gene. *AVR1-CO39* of *M. oryzae* is another avirulence gene involved in host-specificity. The transfer of *AVR1-CO39* to a rice-pathogenic isolate resulted in transformants unable to cause disease on the rice cultivar CO39, while the rice cultivar 51583 that lacks the resistance gene *Pi-CO39(t)* could still be infected [97].

In other cases, lacking complementation of deleted virulence genes by orthologous genes from related species suggested a role in host-specific virulence. For example, the *SIX1* gene of *F. oxysporum* f. sp. *lycopersici* (Fol) was found to be necessary for full virulence of Fol on susceptible tomato but was also recognized by the I-3 resistance gene, leading to avirulence on I-3 tomato lines [98,99]. A homolog of *SIX1* was found to be responsible for virulence of *F. oxysporum* f. sp. *conglutinans* (Foc) on cabbage [100]. Interestingly, virulence of the Foc-*SIX1* deletion mutant on cabbage could be restored by reintroduction of *SIX1* of Foc but not of Fol. This suggested a host-specific virulence role for Fol-*SIX1* [100]. Similarly, virulence of mutants of the wheat pathogen *Zymoseptoria tritici* carrying deletions in the *Zt80707* or the *Zt89160* virulence genes, could be complemented with the respective genes from *Z. tritici*, but not with the respective orthologs from *Zymoseptoria pseudotritici* or *Zymoseptoria ardabiliae* [101] suggesting that the identified genes are involved in host-specific disease development.

In fungi where host specificity is governed by host-selective toxins, validation was done by heterologous expression of the toxin biosynthesis gene in a related species. For example, in the strawberry pathotype of *A. alternata*, transformation-mediated loss of the 1.05-Mb conditionally dispensable chromosome encoding all known toxin biosynthesis genes led to non-pathogenicity [9], which validated the role of the dispensable chromosome in host-specific pathogenicity. In *Alternaria citri*, mutation of a gene encoding an endo-polygalacturonase led to a reduction in virulence, while virulence was unchanged in mutants of *A. alternata* rough lemon pathotype lacking the same gene [102]. This showed that a cell-wall degrading enzyme contributes to virulence of one but not of the other pathotype. In contrast, PbToxB of the bromegrass pathogen *Pyrenophora bromi*, a homolog of the known host selective toxin PtrToxB from the wheat pathogen *Pyrenophora tritici-repentis*, was unexpectedly shown not to be toxic on bromegrass but on wheat, indicating that *P. bromi* has the potential to become a wheat pathogen [103].

These few examples and the surprises they contained illustrate the importance of validation experiments for genes suspected to have a role in host-specificity. Without knowledge of their contribution to host-specific virulence, it is impossible to generate meaningful hypotheses about potential mechanisms.

## 8. Deciphering Functional Mechanisms of Host-Specificity

Knowing the mechanisms that govern host-specific virulence capacities is the ultimate goal in plant-pathogen interaction research. The understanding gained allows the generation of novel plant-protection strategies and ever more reliable prediction of the danger of a particular fungus being involved in future disease outbreaks in novel hosts. In a few cases, more than just the virulence/avirulence-causing gene of the pathogen is known, which allows a better understanding of the basic mechanisms involved in host-specific interactions.

In *P. infestans*, virulence towards potato was shown to depend on the function of the RXLR effector AVR3a in inhibiting enzyme activity of the host ubiquitin proteasome system (UPS) [104]. When studying host adaptation of *P. infestans* and its *Mirabilis jalapa*-infecting sister species *P. mirabilis*, a single amino-acid polymorphism was identified in the host protease and a corresponding single amino-acid change in the pathogen effector as being responsible for virulence of their respective host plants, explaining ecological diversification [105]. In the case of *A. alternata*, the contribution of toxins to host-specific virulence success has been thoroughly proven. Unfortunately, this does not yet explain why a particular toxin would allow virulence only on a particular host plant. For the ACR toxin necessary for virulence of the *A. alternata* rough lemon pathotype, part of the functional mechanism was unraveled. Toxicity on rough citrus (*Citrus jambhiri* Lush.) was found to depend on the differential post-transcriptional processing of transcripts of the mitochondrial *ACRS* (ACR-toxin sensitivity) gene, which is present in both toxin-sensitive and toxin-insensitive citrus but processed to shorter transcripts in mitochondria of insensitive plants [106]. This work showed that host specificity of the rough lemon pathotype of *A. alternata* towards its host is due to altered mitochondrial RNA processing. In *Alternaria brassicicola*, that causes black spot on *Brassica* plants, the essential contribution of the AB-toxin to virulence is well known. It was shown that a host-derived factor, an oligosaccharide of 1.3 kDa, secreted from the plant just after *A. brassicicola* spore germination, was necessary to induce AB-toxin production [107] showing the involvement of a host-derived factor in the production of host-selective toxins.

## 9. Conclusions

A plethora of methods has been developed to help in the identification of host-specificity factors. While most molecular methods are excellent for describing molecular differences between host-selective strains, few are suited for rapid comparative identification of genes involved in host-specificity. With the development of next-generation sequencing technologies, at least the relatively rapid prediction of potential target genes now becomes possible. However, prediction alone is not enough. Without validation of functional involvement in host-specificity, the underlying functional mechanisms cannot be resolved. Resolving functional mechanisms is still a slow and challenging task, involving rigorous scientific work and extensive experimentation.

In most cases, in spite of great progress in the description of molecular differences of host-specific strains and in the prediction of genes possibly involved in host selection, we are still far from understanding how host-specific infection is achieved. What is the reason for this apparent lack of progress? Are the available methods insufficient? Do researchers just not search hard enough? Rapid identification of host-specificity factors could be hampered by the specific characteristics of the investigated system. So far, host-specificity factors have been identified only in systems where host-specificity depended on a single gene. However, this might not represent the majority of the cases, and adaptation might depend on more than just the presence of one gene or one altered amino acid in the other systems. In a lot of cases, secreted fungal proteins were suspected to modulate interaction with the plant and be the reason for being able to spread only on a specific host plant. However, this might not be a general solution. Most genes identified as being under positive selection pressure in the two *formae speciales* of *S. reilianum* encoded proteins that are internal to *S. reilianum* [108], suggesting that the capacity to proliferate successfully on a specific host might depend on adapted metabolic capacities. On the other hand, it could be that host-specific differences are explained not by the presence of particular genetic markers in the host-specific strains but are dependent on host-specific gene expression. In this case, identification of host-specificity factors will likely not be successful if considering only genome sequences for comparison, and gene-expression changes may need to be considered. Host-specific changes of fungal gene expression might not even be encoded in the pathogen genomes. As was shown in the *B. cinerea*-*Arabidopsis thaliana* interaction, small RNAs of the plant may migrate into the pathogen to regulate fungal gene expression [109]. If this turns out to be a general principle, most of the molecular comparative studies aimed at identifying host-specific molecular differences in the fungal pathogens are doomed to fail.

In spite of significant progress, the battle for understanding the principles of host selection in fungal plant–pathogen interaction is not yet won. A variety of comparative methods is available to help in the identification of host-specificity factors. An intelligent combination of classical genetics and next-generation sequencing in the consideration of gene-expression changes of both the pathogen and host may be needed in order to unravel the mechanistic basis of host-specificity of plant-pathogenic fungi.

## Figures and Tables

**Table 1:** Method-specific advantages and disadvantages of molecular methods typically used for comparison of host-specific fungal isolates.

Method ^1^	Advantages	Disadvantages
Restriction fragment-length polymorphism(RFLP)	Can detect allelic variants	Large DNA quantity needed, typically only 1–3 loci detected, usually radioactive labeling is used
Random amplified polymorphic DNA (RAPD)	Faster than RFLP, less DNA is needed, can detect 1–10 variant loci, suitable for detection of broad scale genetic structural differences	Cannot detect allelic variants (heterozygous alleles or homologous alleles normally give the same result), less reliable, polymerase chain reaction (PCR)-dependent assay
Simple sequence repeats; microsatellites (SSR)	More accurate than RAPD, suitable for discriminating different subpopulations	Microsatellite markers may not be evenly distributed in the genome, SSR are located in non-coding regions, false alleles or null alleles may be detected due to technical artifacts, blurry bands may occur
Amplified fragment-length polymorphism (AFLP)	Combines benefits of RAPD and RFLP	Difficult to develop locus-specific marker (fragment) proprietary technology to score heterozygous and homozygous
Analysis of mitochondrial DNA (mtDNA analysis)	Powerful tool for studying inheritance of mitochondrial genomes, for phylogenetic and population genetic analysis, for species identification and barcoding	In uniparental-mtDNA inheritances, no information about other parent: should be coupled with genomic-DNA analyses. In case of mtDNA recombination (bi-parental inheritance) many analysis not doable
Sequencing of internal transcribed spacer regions (ITS sequencing)	ITS1 and ITS2 regions are species-specific and have large copy numbers, ITS sequencing can be used in metagenomics studies (meta-barcode), can be coupled with NGS technique	Limited to discriminate intra- and intergeneric species
Analysis of protein abundance of all proteins (proteomics)	Many different techniques available, e.g., two-dimensional electrophoresis coupled to mass spectrometric protein identification, can analyze vast array of proteins at once, can do high throughput, high sensitivity possible, relative as well as absolute protein abundance quantification possible	Each technique has its own limitation, not all proteins can be identified by one single method. Results may be tissue- and environmental condition-dependent
Sequencing using next-generation sequencing techniques (NGS sequencing)	Identify millions of single nucleotide polymorphisms (SNPs) as well as insertions and deletions (INDELs) at once	PCR-born false variants, data analysis needs bioinformatic know-how and computing power

^1^ See text for a description of the respective method.
